# A Scalable Perfusion Culture System with Miniature Peristaltic Pumps for Live-Cell Imaging Assays with Provision for Microfabricated Scaffolds

**DOI:** 10.1089/biores.2015.0024

**Published:** 2015-08-01

**Authors:** Sreenath Balakrishnan, M.S. Suma, Shilpa R. Raju, Santosh D.B. Bhargav, S. Arunima, Saumitra Das, G.K. Ananthasuresh

**Affiliations:** ^1^ Center for Biosystems Science and Engineering, Indian Institute of Science, Bengaluru, Karnataka, India.; ^2^ M^2^D^2^ Laboratory, Department of Mechanical Engineering, Indian Institute of Science, Bengaluru, Karnataka, India.; ^3^ Saumitra Das's laboratory, Department of Microbiology and Cell Biology, Indian Institute of Science, Bengaluru, Karnataka, India.

**Keywords:** 3D printing, bioreactor, live imaging, microfabricated scaffolds, perfusion culture, peristaltic pump, VeroWhite

## Abstract

We present a perfusion culture system with miniature bioreactors and peristaltic pumps. The bioreactors are designed for perfusion, live-cell imaging studies, easy incorporation of microfabricated scaffolds, and convenience of operation in standard cell culture techniques. By combining with miniature peristaltic pumps—one for each bioreactor to avoid cross-contamination and to maintain desired flow rate in each—we have made a culture system that facilitates perfusion culture inside standard incubators. This scalable system can support multiple parallel perfusion experiments. The major components are fabricated by three-dimensional printing using VeroWhite, which we show to be amenable to *ex vivo* cell culture. Furthermore, the components of the system can be reused, thus making it economical. We validate the system and illustrate its versatility by culturing primary rat hepatocytes, live imaging the growth of mouse fibroblasts (NIH 3T3) on microfabricated ring-scaffolds inserted into the bioreactor, performing perfusion culture of breast cancer cells (MCF7), and high-magnification imaging of hepatocarcinoma cells (HuH7).

## Introduction

Some of the emerging research areas in contemporary cell biology are studies on single live cells using microscopy, cell culture on three-dimensional (3D) scaffolds, and studies of response of a cell to chemical and mechanical stimulants such as drugs, fluid shear, etc. All these studies require specialized systems to serve the diverse needs such as cells grown on cover-slips for high-magnification imaging, flow chambers for effecting fluid flow, etc. Most of these requirements are not catered to by conventional cell culture systems, notably Petri dishes and multiwell plates. While it is possible to insert a scaffold to grow cells inside Petri dishes, live high-resolution imaging at sufficiently high magnification or perfusing of media are not possible with them. On the other hand, conventional systems are extremely convenient to handle especially being amenable for addition and removal of cells or chemicals individually by pipetting. They fit inside standard incubators.

While there are many live-cell chambers available,^1–4^ we are not aware of any system that can be used conveniently inside an incubator as well. Similarly, there are perfusion culture systems that can do convenient perfusion culture with scaffolds,^3,5–8^ but they are not adaptable to live-cell studies. Many of the microfluidic approaches can control the microenvironment of the culture very precisely (reviewed in Halldorsson et al.^9^ and Young and Beebe^10^), but they are not usually as convenient as normal cell culture techniques and are not adapted for live cell imaging at high magnifications. Hence, we wanted to design a generic cell culture system consisting of bioreactors that meet the aforementioned requirements while not compromising the ease of handling.

For bioreactor to be perfusion ready, it should be a watertight flow chamber. Most high-resolution objectives (oil immersion) are designed for imaging across a cover-slip. It should be noted that the scaffolds can be microfabricated on cover-slips. Hence, we made the bioreactor such that cover-slips can be conveniently incorporated inside them so that cells are grown on these cover-slips. For convenience of operation, it should be possible to pipette liquid in and out of the bioreactor and it should be of sizes similar to standard cell culture dishes. The bioreactor was hence designed to have a removable top to facilitate addition and removal of cells/chemicals and still be of the size of a well in a standard 24-well plate.

One of the disadvantages of using cover-slips in a flow chamber is that the system becomes very sensitive to the force that needs to be applied on the cover-slip to seal it to be watertight. Any decrease in the force may lead to leakage while large forces may break the cover-slip. These issues are even more serious in perfusion culture systems where multiple such bioreactors are connected in series because leakage in any one of them can upset an entire experiment.

Most of the commercially available peristaltic pumps used to set up perfusion of culture medium are bulky (20–30 cm on the side and 3–5 kg mass). The pump is typically kept outside the incubator with the tubes from the pump entering and exiting the incubator. Using multiple pumps for the same incubator requires more tubes to be taken into the incubator. This is cumbersome and results in the incubator door being ajar. To solve this problem and to be able to perform multiple parallel perfusion experiments, we have designed miniature peristaltic pumps that can reside completely inside the incubator and perfuse medium into the bioreactors.

The bioreactor and the casing of the peristaltic pump were fabricated using 3D printing out of VeroWhite by PolyJet printing technology (www.stratasys.com). Our experiments show that this material is amenable for use to make cell culture dishes even with cell culture media coming into contact with them. Since 3D printing can build virtually any design easily, this opens many opportunities for designing bioreactors for a variety of needs. Further, this material can withstand multiple sterilization cycles (washing with ethyl alcohol and exposure to ultraviolet [UV]) and autoclaving. While some bioreactors do get deformed, they do so after more than 20 or so sterilization and autoclaving cycles.

The bioreactors and the peristaltic pumps are conveniently placed on an assembling plate containing a pump-control circuit board common for all peristaltic pumps and separate media reservoirs for each bioreactor. Only an electrical connection to power the control circuit needs to come out of the incubator. A previously reported perfusion system in a multiwell format^5^ that goes totally inside the incubator and uses compressed air for actuating perfusion pumps has been a motivation for designing our system. In our experience, we have not found many biology laboratories equipped with compressed air lines for using as an actuation mechanism. The authors^5^ stated that electrically driven pumps cannot sustain for long inside the harsh conditions of the incubator (temperature of 37°C and 100% relative humidity), but in our experience the pumps have worked without problems for around two weeks, which is the maximum that we have driven them continuously. While our system probably cannot sustain as long as theirs (6 months of continuous operation inside the incubator) we expect the pumps to work at least for 2–3 weeks. Hence, our system is currently suitable for such medium-term cultures lasting around 2–3 weeks.

We show the adaptability of the system to various cells and situations by demonstrating the growth of primary rat hepatocytes in the bioreactor, live imaging of NIH 3T3 fibroblasts on microfabricated ring scaffolds, growth of breast cancer cells (MCF7) under perfusion, and high-magnification live imaging of liver carcinoma (HuH7) cells.

## Design and Fabrication

### Miniature perfusion bioreactor

**FIG. 1.**
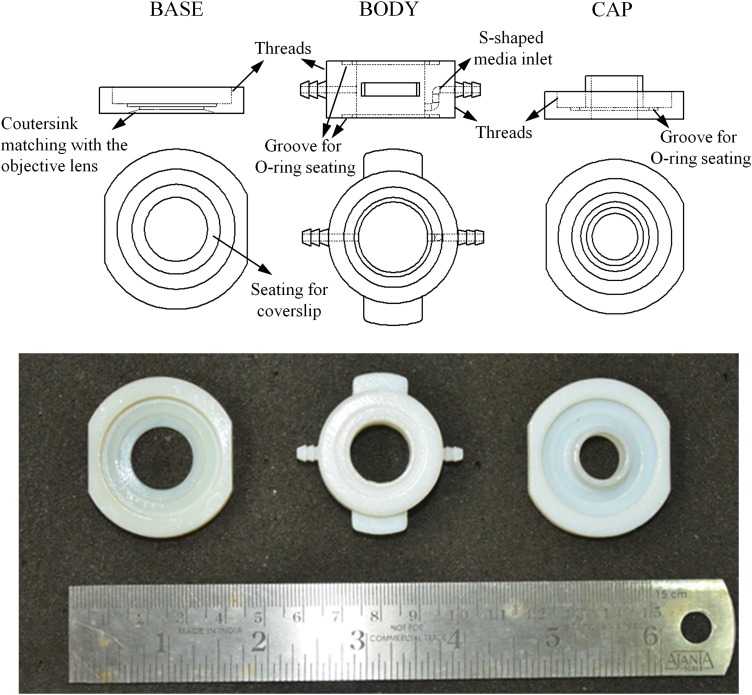
Design of the bioreactor. Three parts of the bioreactor namely base, body, and cap. The bottom of the base has a countersink which matches the highest magnification objective lens and also has a seating for the cover-slip. The base has internal threads which match the external threads on the body. The body has two grooves on the bottom and top for seating the silicone O-ring. The media inlet is S-shaped so that the media enters the bioreactor at a height lower than that at which it exits. The external thread on the body matches the internal threads on the cap. The cap too has a groove for seating the silicone O-ring and a cylindrical protrusion which will be filled with polydimethylsiloxane (PDMS).

**FIG. 2.**
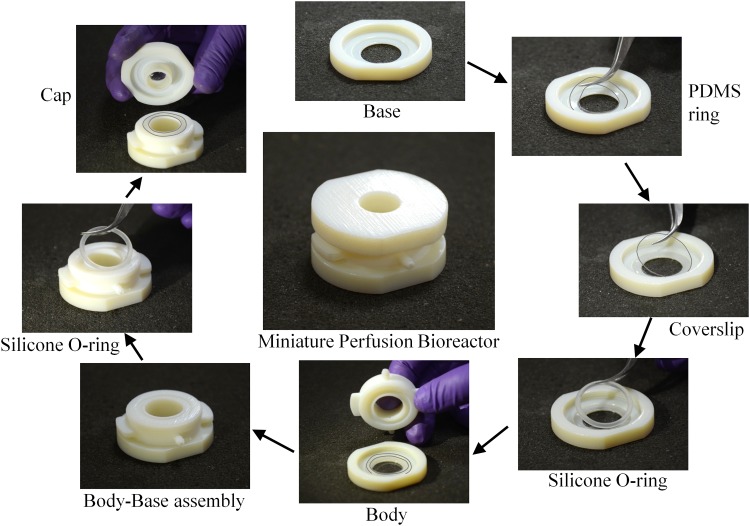
Assembling the bioreactor. A thin PDMS ring is placed on the seating made for the cover-slip on the base. A cover-slip is then placed on the PDMS ring. Further, a silicone O-ring is placed on top of the cover-slip. The body is then screwed on to the base with due care to make sure that the silicone O-ring sits in the groove meant for it on the bottom of the body. Another silicone O-ring is placed on top of the body and the cap is screwed on top with the silicone O-ring sandwiched between the grooves on both the body and the cap.

The bioreactor is made in three parts, namely, a base, a body, and a cap (Fig. 1). Since we required that it should be possible to pipette liquid into and out of the bioreactor, we decided to incorporate a cap that can be easily removed and secured. For ease of insertion and removal of cover-slips from the bioreactor, we decided to incorporate a base and a body between which the cover-slip will be tightly secured. All these parts were designed in SolidWorks (www.solidworks.com) and made out of VeroWhite (www.stratasys.com) by 3D printing (Object 260 Connex). The cover-slip is placed on the base and the base is screwed onto the body. A silicone O-ring is used as a gasket to obtain a watertight joining of the cover-slip with the body. A thin polydimethylsiloxane (PDMS) layer is placed below the cover-slip, that is, on the base, to give cushioning for the cover-slip when it is pressed between the body and the base. The cap is then screwed onto the body with a silicone O-ring gasket to obtain a water-tight flow chamber as can be seen in Figure 2. The cap is filled in the middle with PDMS to allow for exchange of gases and for letting light in for microscopy.

The base has a seating for the cover-slip and the bottom of the base has a countersink (Fig. 1), which matches the profile of the highest magnification objective lens (in our case, Leica 40×OIL 1.3 NA), so that the objective can reach close to the cover-slip and have a large area where things can be focused. The base has an internal thread (pitch 1 mm) that matches the external threads on the body. The bottom of the body has a circular groove (Fig. 1), which eases the seating of the silicone O-ring. The silicone O-ring is pressed between the cover-slip and this groove on the body and this makes the joint between the cover-slip and the body watertight. Since cover-slips are very delicate, securing the body with the base should be done very carefully. If it is not tightened enough, the bioreactor will leak and excessive tightening will break the cover-slip.

The media inlet on the body is S-shaped so that the medium enters the bioreactor at a height lower than the height at which it exits. This helps in enhanced mixing of the media inside the bioreactor and reduces dead zones inside the bioreactor. We have also observed that at least three threads are required to avoid automatic loosening of the joints. This constrains the height of the media inlet and outlet ports since these ports have to be above the threads and there should be enough space between the ports and the base and the cap for conveniently securing the tubes for media perfusion. The media inlet and outlet ports are designed with serrated edges so that the tubing from the pump can be tightly secured to the body. The body has two holding pads in a direction orthogonal to the media inlet and outlet for convenient handling, especially while screwing the body to the base and the cap. While the internal diameter of the body was made to match that of the standard 24-well plate (16 mm diameter for each well), the external dimensions were decided for ease of handling. The upper part of the body has a circular groove for giving a good seating to the silicone O-ring and an external thread which matches the internal threads on the cap (pitch 1 mm). The cap has a matching groove for seating the silicone O-ring, and the hole at the end of the cap that goes into the body is filled with a PDMS layer of about 2 mm thickness.

All the components that go into the bioreactor can be autoclaved and hence can be sterilized easily and reliably. After each culture, the cover-slip can be discarded and the rest of the components can be reused after sterilization by washing with 70% ethanol and deionized water, followed by autoclaving.

**FIG. 3.**
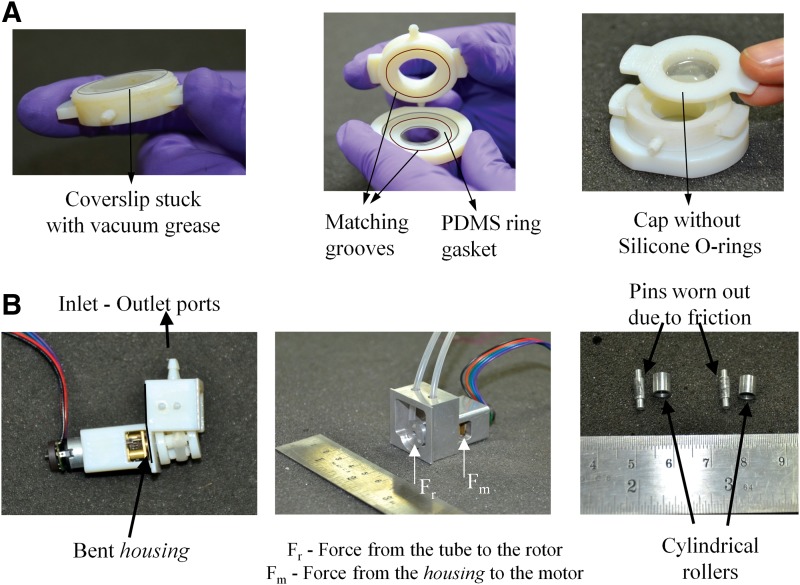
Initial bioreactor and peristaltic pump designs. **(A)** Initial bioreactor designs. Left: The design did not have a base and the cover-slip was just stuck to the body using vacuum grease. Center: A PDMS ring gasket was used for sealing by placing it on top of the cover-slip with matching protrusions and grooves on the base and body, respectively. Right: The cap was just screwed on top of the body without any silicone O-rings for sealing. **(B)** Initial peristaltic pump designs. Left: A modular peristaltic pump with inlet and outlet ports. The tube is connected between the inlet and outlet ports on the inside of the housing (not shown). The housing is bent because of the force exerted by the tube on the rotor. Center: Housing and rotor made out of metal to avoid the bending of the housing and wearing out of the rotor. The force exerted by the tube on to the rotor and the support force exerted by the housing on the motor is also shown. Right: Pins and cylinders used as rollers on the rotor. The wear on the pins due to the friction during operation of the pump is shown.

The aforesaid design has been refined and redesigned based on the problems faced in our first few prototypes. Our first prototype had just two pieces, the body and the cap with the cover-slip stuck to the bottom of the body using vacuum grease (Fig. 3A, left). During perfusion inside the incubator, the cover-slip would get detached from the body and the media would leak out of the bioreactor. Our second prototype had the base screwed on to the body, but had a custom-built PDMS ring as a gasket (Fig. 3A, center). A ring protrusion was made on the base with a matching groove on the body and the PDMS ring gasket was secured between these features. The PDMS ring gasket could not give sufficient tight fit and was replaced by a silicone O-ring in the next prototype. This prototype worked well for a few trials, but multiple usages with loosening and tightening of the cap reduced the tight fitting of the body with the cap leading to a leak from the top (Fig. 3A, right). Hence, an additional silicone O-ring gasket was incorporated between the cap and the body.

### Miniature peristaltic pump

**FIG. 4.**
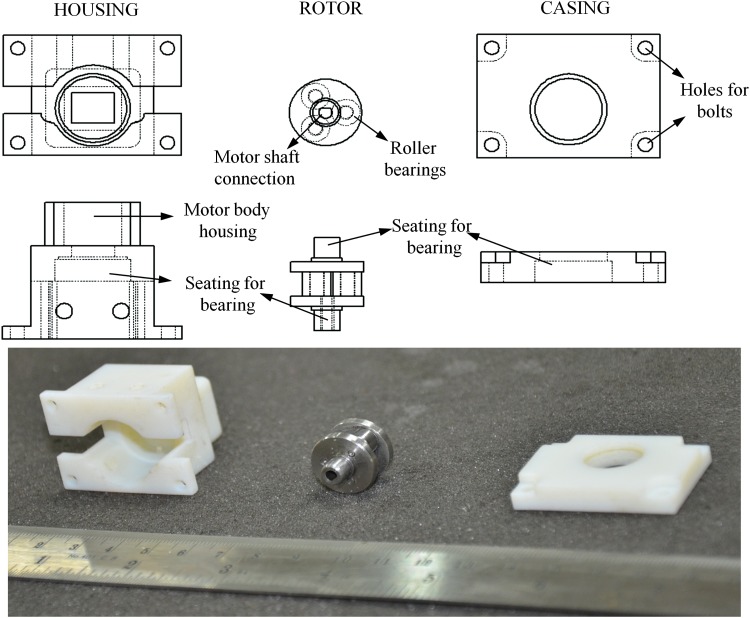
Design of the miniature peristaltic pump. The peristaltic pump has three main parts—housing, rotor, and casing. The housing holds the motor and has a seating for the ball bearing, which holds the rotor in place. The rotor has three needle bearings, which act as rollers. The needle bearings roll on pins that are held between two flanges. The flange which attaches to the motor shaft has a hole that matches the profile of the shaft. The rotor is held on its other end by another ball bearing that sits on the seatings of the rotor and the casing. The casing is secured on to the housing using four bolts.

The miniature peristaltic pump that we have designed is around 20–30 mm in size and weighs around 70 g. The peristaltic pump was designed in three parts, namely, housing, rotor, and casing (Fig. 4). The parts were designed in Solidworks and the housing and casing were made out of VeroWhite using a 3D printer while the rotor was made of stainless steel. The housing holds the motor, the tubes, and has a cylindrical surface onto which the tubes are pressed by the rotor. The rotor is assembled out of three parts, namely, flanges, shafts, and needle bearings. The needle bearings act like rollers, which press the tubes onto the cylindrical surface on the housing. These rollers are mounted on the shafts that are held by the flanges. The flange that goes onto the motor shaft has a hole at the center, which matches the diameter of the motor shaft. The rotor is held at both ends by a ball bearing, which is in turn fitted into the housing and the casing. When the rotor is pressing the tube, the tube exerts an equal and opposite force on the rotor, which is transferred to the housing and the casing (but not to the motor body) by these ball bearings. The casing holds the rotor through a ball bearing and is screwed onto the housing using four bolts.

**FIG. 5.**
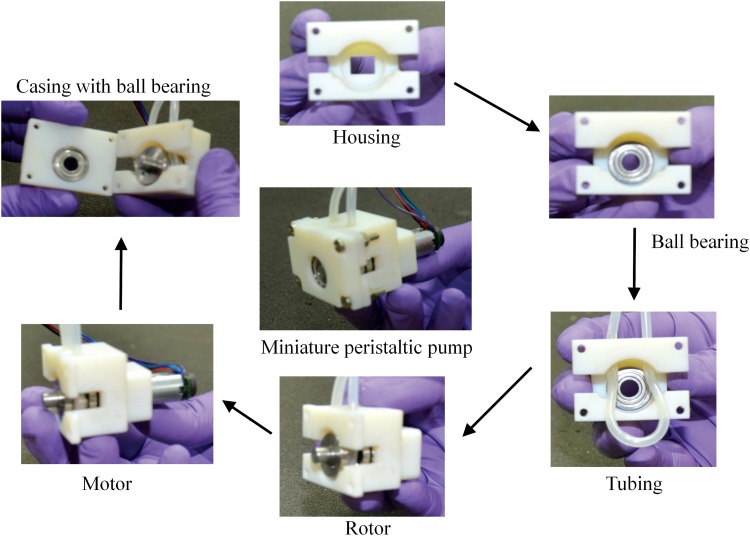
Assembling the miniature peristaltic pump. A ball bearing is press fitted on to the seating on the housing. The tubings are then inserted through the two holes on the top of the housing. The rotor is then carefully press fitted into the ball bearing inside the housing such that the tubing is pressed between the rotor and the housing. Further the motor is fixed from behind the housing such that the motor shaft inserts into the flange on the rotor. Another ball bearing is press fitted to its seating on the casing and the casing is then fitted on the housing such that the rotor is held by the ball bearing. The casing is secured on the housing using four bolts.

The assembling procedure for the pump is shown in Figure 5. First, the ball bearing is inserted into the housing and press-fitted onto the seating shown in Figure 5. Next, the tube is inserted into the housing through the holes on the upper part. The rotor is then inserted such that the seating on the rotor is press-fitted on to the bearing on the housing and the tube goes around the roller and is pressed on the housing. Next, the motor is inserted from behind the housing through the motor body and fitted onto the rotor. Further, another ball bearing is press-fitted onto the casing and the rotor. Finally, the casing is secured to the housing by four bolts.

Some of our previous prototypes are shown in Figure 3B. The first prototype used the idea of a modular peristaltic pump design wherein the pump had an inlet and outlet port to be connected to the tube coming from the media reservoir and going to the bioreactor, respectively. The advantage of peristaltic pumps over other pumps for cell culture is that the medium comes into contact only with the tube and not with any part of the pump. Such a modular design would entail media coming into contact with the inlet and outlet ports of the pump and would negate this advantage. Further, the housing of the pump was bending and the rotor was getting worn out since it was made out of VeroWhite, a 3D printer material (Fig. 3B). Hence, in our next design, we made the housing and rotor out of stainless steel to avoid the bending of the housing and wearing of the rotor. In this design, the force from the tube onto the rotor was transferred to the motor body. Any clearance between the motor body and the housing resulted in the rotor oscillating up and down. When we made the motor to fit tightly with the housing, it became difficult to insert the motor into the housing. Hence, we decided to avoid transfer of forces from the rotor to the motor by supporting the rotor by ball bearings inserted into the housing. Further, we changed the rollers on the rotor from a cylinder around the pins to needle bearings to avoid wearing of the pin and roller due to friction.

### Perfusion culture setup

**FIG. 6.**
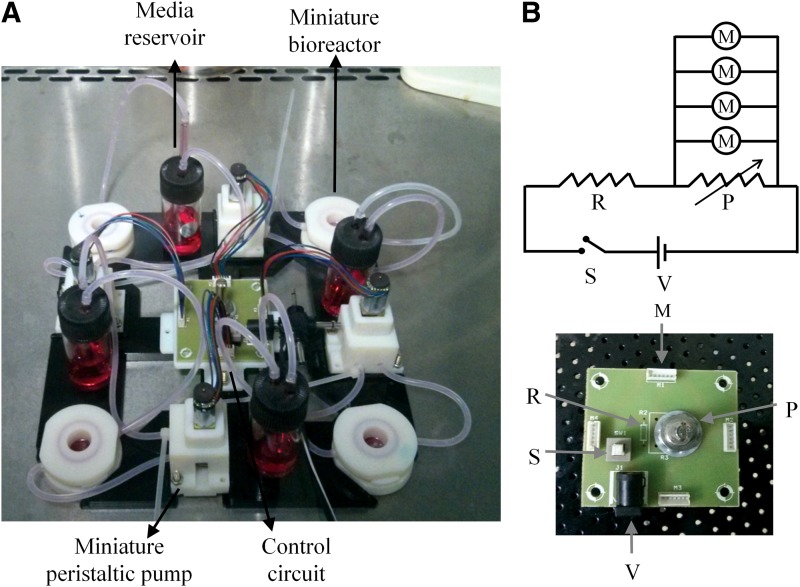
Perfusion culture setup and the pump control circuit. **(A)** An assembly plate for securing the bioreactors, peristaltic pumps, media reservoirs, and the electric control circuit. A control circuit placed at the center draws power from an external source and drives four pumps. Tubes are routed from the reservoir to the pump and then to the bioreactor. The outlet of the bioreactor drains back into the reservoir. **(B)** The pump control circuit essentially consists of a potentiometer (P) across which several motors (M) are connected. Another resistor (R) is connected in series with the potentiometer to avoid shorting of the circuit case when the potentiometer is set to zero resistance. There is also a switch (S) in series with the resistor and the potentiometer for conveniently powering off the circuit.

An assembling setup was envisaged for performing multiple perfusion experiments using the bioreactors and peristaltic pumps. We wanted to be able to perform multiple parallel experiments and hence designed the system to have multiple parallel perfusion cultures each with a bioreactor connected to a peristaltic pump and media reservoir of its own (Fig. 6A). The assembling plate was designed in Solidworks and 3D printed (Objet260 Connex). The pumps were connected to a common circuit board, which provides constant voltage to all the motors and hence drive them at equal flow rates. The board was driven by externally supplied DC voltage using an AC to DC converter adapter. The entire assembly with the bioreactors, pumps, and motors can be taken inside the incubator and only the electric wire of the adapter needs to come out of the incubator. Glass containers (BOROSIL autoclavable 15-mL capacity) were used as media reservoirs. Two holes were drilled on the cap of the container and the glass tubes (BOROSIL autoclavable) were inserted into the holes. The gap between the holes and the glass tube was sealed using PDMS to avoid any particles from falling into the reservoir. The dimension of the glass tube was chosen such that the tubings (inlet to the pump and the outlet from the bioreactor) fit the glass tube tightly. Further, a sterile syringe filter (pore size 0.2 μm) was connected to the outlet of the pump to avoid any particulates from perfusing into the bioreactor. The tubings were connected to the syringe filter using suitably cut-out pipette tips. The media was recirculated from the reservoir through the pump to the bioreactor and back into the reservoir; otherwise the secreted factors will be removed completely.^11,12^ However, our system can be reconfigured to avoid recirculation and emptied to another reservoir if it is so desired in an experiment.

The control circuit consists of a potentiometer and the motors connected in parallel to this potentiometer (Fig. 6B). A resistor is connected in series with the potentiometer as a safety mechanism in case the potentiometer is set at zero resistance. The voltage across the motors (and thereby the pump flow rates) can be adjusted using the potentiometer. The voltage across the motors can be calculated using Kirchhoff's law.^13^

\begin{align*}V_M = \frac { \left( \frac { PR_M }  { 4P + R_M } \right) }  { \left( R + \frac { PR_M }  { 4P + R_M } \right) } V \tag { 1 } \end{align*}

where *P* is the potentiometer resistance, *R* the safety resistor resistance, *R_M_* the motor resistance, *V* the applied voltage, and *V_M_* the voltage across the motors. From Equation (1), it can be seen that the voltage across the motors varies nonlinearly with the potentiometer resistance. Further, the voltage across the motors changes when we remove or add motors to the circuit. This is a minor inconvenience, as the voltage across the motor needs precalibration for various positions of the potentiometer for each motor.

The present system can accommodate four parallel perfusion experiments and it is scalable. The only constraints on the scale are the size of the incubator and the limitations in handling many experiments. Since the perfusion cultures share only electrical connections, it is very clear that the system can simultaneously perform parallel experiments with different cell types and chemical stimulants such as drugs.

## Materials and Methods

### Perfusion cell culture

The bioreactor parts, base, body, and cap, silicone O-rings, and PDMS gaskets, were ultrasonicated once in 70% ethanol and twice in deionized water for 15 min each. These were then autoclaved and UV sterilized overnight inside a laminar air flow hood. The cover-slips were cleaned using detergent (3% Extran solution) and further with 70% ethanol for 1 h at 80°C each and then ultrasonicated thrice with deionized water for 15 min each followed by drying overnight at 80°C. The cover-slips were then sterilized using UV radiation for over 24 h. The bioreactors were assembled inside a laminar flow hood maintained in sterile conditions. The cover-slips in the bioreactors were coated with collagen by adding 700 μL of 30 μg/mL of type I collagen (rat tail, Gibco by Life Technologies) for 4 h at 37°C. Excess collagen is removed from the bioreactor and the bioreactor is thoroughly washed with phosphate-buffered saline (PBS) before seeding cells. Cells were maintained in Dulbecco's modified Eagle's medium (DMEM)—high glucose (Sigma–Aldrich) and harvested at 90% confluency. Around 30,000 cells were seeded into the bioreactor and they were allowed to attach for 24 h before starting perfusion.

The pump parts-housing, casing and pump tubings, were ultrasonicated once in 70% ethanol and twice in deionized water for 15 min each. They were then autoclaved and sterilized using UV radiation overnight. The pump parts, which could not be autoclaved, namely the rotor, ball bearings, and motor were thoroughly cleaned with 70% ethanol and sterilized using UV radiation overnight. The assembly plate and the circuit were also thoroughly cleaned with 70% ethanol and sterilized using UV radiation overnight. Media reservoir with the attached glass tubes were ultrasonicated once in 70% ethanol and twice in deionized water for 15 min each, autoclaved, and then sterilized using UV radiation overnight.

The pump was assembled inside a sterile laminar air flow hood along with the assembly plate and the printed circuit board. Before starting perfusion, the tubings were perfused with tissue culture grade autoclaved water at high flow rates under UV radiation for 2 h and further given one round of media wash. Sterile syringe filters were then connected to the pump outlet. The outlet from the syringe filter was connected to the bioreactor inlet and the outlet from the bioreactor was connected to the media reservoir. Perfusion was started inside the sterile laminar air flow hood and the pump flow rate was set to 0.6 mL/min. Perfusion was allowed to stabilize for around 5 min before transferring the entire setup into an incubator maintained at a temperature of 37°C and 5% CO_2_.

### Estimation of shear stress on cells using computational fluid dynamics

The shear stress on the cells induced by fluid flow was estimated using computational techniques. The fluid velocity was obtained by numerically solving for Navier–Stokes equations on the fluid inside the bioreactor:

\begin{align*}\rho ( {\bf u} \nabla ) {\bf u} = - \nabla p + \eta \nabla^2 {\bf u} \tag{2}\end{align*}

where $\rho$ is the density of the media (1,000 kg/m^3^),^14^ **u** the velocity vector, *p* the pressure, and $\eta$ the viscosity of the media (0.6915×10^−3^ Pa·sec).^14^ The media is assumed to be an incompressible and Newtonian fluid. Other assumptions like no-slip boundary condition on the boundaries, uniform velocity at the inlet of the bioreactor, and zero gauge pressure at the outlet of the bioreactor were made. Flow inside the bioreactor was assumed to be laminar. The flow from the pump was assumed to be uniform and any pulsatile action was neglected.

The numerical simulation was done using the Laminar Flow module in COMSOL 4.2 (COMSOL AB). The geometry was created, meshed, and solved using COMSOL and the resultant shear stress on the bottom of the bioreactor was calculated and plotted.

### Growing MCF7 cells under perfusion

Around 30,000 MCF7 cells were seeded in the bioreactors and controls. The cells were allowed to attach for 24 h before starting perfusion. The cell viability on the second, fourth, and sixth days were estimated by using Calcein AM–Ethidium Bromide live–dead assay kit (Life Technologies). The days are counted from the day on which cells were seeded. Fluorescent images of a large portion (∼80% of the total area) of the bioreactors (static and perfusion) were obtained by using the tile scan option on a fluorescent microscope with a motorized stage. The live and dead cells were manually counted on a set of 10 images which were randomly selected from the total number of images for each tile scan. The total number of cells in the bioreactors was extrapolated from the average of the number of cells in these 10 images by multiplying with the ratio of the area of the bioreactor to the area of the image. Cell viability in 24-well plates and 24-well plates coated with collagen was also estimated to infer the contribution of collagen to cell growth and viability.

### Live imaging of cell growth on scaffolds

Scaffolds used were rings of 300 μm inner diameter and about 5 μm height. Scaffolds were made on a 22 mm circular cover-slip using photolithography using SU-8, a negative photoresist used in lithography-based patterning in microfabrication. The cover-slips were heated to about 200°C for 2 min to evaporate water that might be there on the cover-slip and allowed to cool to room temperature. The cover-slip was spin-coated with SU-8 2005 at 4,000 rpm for 40 sec to get a uniform layer of SU-8 of thickness 5 μm over the cover-slip. The cover-slip was then prebaked at 95°C for 10 min to ensure that SU-8 does not stick to the mask during UV exposure. Further, the cover-slip was exposed to UV using an EVG 620 Mask Aligner with a peak exposure energy of 374 mJ/cm^2^. After the exposure, the cover-slip was postbaked at 95°C for 2 min. The postbaked cover-slip was then immersed in SU-8 developer solution for 7 min to remove the unexposed regions and further dehydrated at 200°C for 2 min to get a dry sample.

The scaffold was then washed thoroughly with PBS and sterilized by exposing to UV overnight. The cover-slip containing the scaffold was assembled into the bioreactor and further coated with collagen by immersing it in a solution of 30 μg/mL of type I collagen (rat tail, Gibco by Life Technologies) for 4 h at 37°C to aid cell attachment. NIH 3T3 cells were added into the bioreactor containing the scaffold immediately after collagen coating and allowed to attach for 24 h.

The bioreactor was transferred to a microscope with a live-cell stage for long-duration live imaging. The temperature and CO_2_ were set to 37°C and 5%, respectively, and allowed to stabilize for 5 min before inserting the bioreactor. Various locations on the scaffolds were marked and pictures of these marked locations were taken every 20 min for 40 h.

### Primary rat hepatocyte isolation and culture

Primary rat hepatocytes were isolated from normal rat (Wistar strain) by collagenase perfusion.^15^ The viability of the cells was checked using Trypan blue (0.4%) exclusion. Only those preparations of hepatocytes with viability greater than 90% were used for the experiments. About 4×10^5^ hepatocytes were seeded in each bioreactor and maintained in DMEM containing 100 μg/mL of penicillin and streptomycin. The media were changed every day. The bioreactors were coated with collagen before seeding cells by incubating the bioreactor with 700 μL of 30 μg/mL of type I collagen (rat tail, Gibco by Life Technologies) for 4 h at 37°C.

### High-magnification live imaging of HuH7 cells

Around 30,000 HuH7 cells were seeded into the bioreactor and allowed to attach and grow for 24 h. The cells were then incubated with the Calcein AM–Ethidium Bromide live–dead assay (Life Technologies) mixture at 37°C for 10 min. The bioreactor was then transferred to an inverted fluorescence microscope (Leica DMI6000 B) and imaged using a high numerical aperture oil immersion objective (HCX PL APO 40×/1.25–0.75 OIL) for around 30 min.

## Results and Discussion

### Shear stress in the bioreactor

**FIG. 7.**
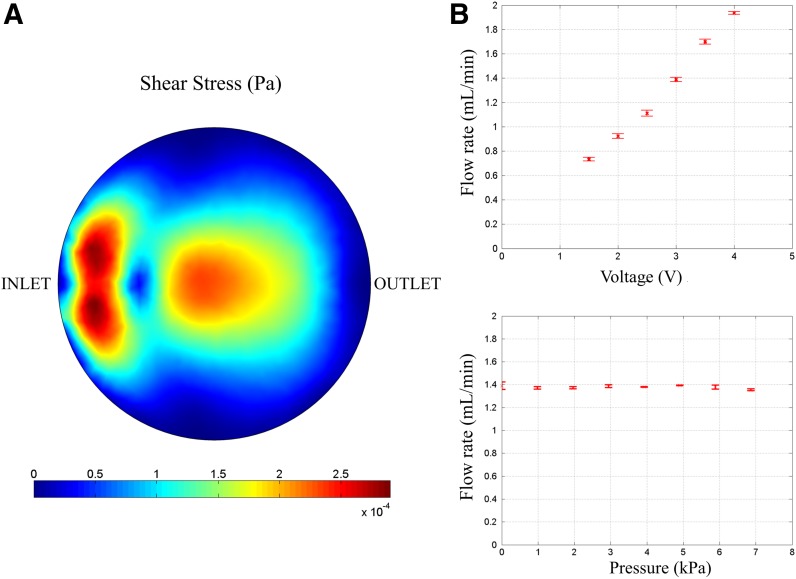
Bioreactor and pump characterization. **(A)** Shear stress in the bioreactor—The total shear stress on the cells due to the flow of media from the peristaltic pump is shown. The maximum shear stress is around 0.29 mPa and occurs close to the inlet. **(B)** Pump flow characterization—Flow rate (mL/min) of the pump at zero pressure head for various voltages (in Volts; top) and at a constant voltage (3*V*) for various pressure heads (in kPa; bottom).

The distribution of shear stress is asymmetric with regions of maximum shear stress occurring close to the bioreactor inlet and further local maxima near to the center of the bioreactor (Fig. 7A). The asymmetry in the shear stress distribution is due to the inlet being at a lower height than the outlet. The maximum shear stress on the cells is around 0.29 mPa, while the reported shear stress level that is harmful for cell viability is about 33 mPa^16^—about three orders of magnitude higher.

### Pump characterization

The flow rate of the pump was measured for different voltages at zero pressure head across the pump and also for different pressure heads at a constant voltage of 3 V across the pump (Fig. 7B, top). The flow rate increases almost linearly with the voltage for zero pressure head. In the case of varying pressure heads (0–7 kPa) at constant voltage of 3 V, the flow rate is almost constant (Fig. 7B, bottom). We have measured the flow rates against pressure heads only up to 7 kPa because when we are perfusing the media across a single bioreactor from the reservoir and back into the same, we do not expect the pressure head to be very large. In fact, the pressure drop across the bioreactor is only around 0.66 Pa as was found from the computational fluid dynamics simulation. Our pump, like most other peristaltic pumps, can work at much higher pressure heads. We have tested it up to a pressure of 2 m high column of water (20 kPa) and the pump worked without any discernible difference in the flow rate.

### Viability of MCF7 cells under perfusion

**FIG. 8.**
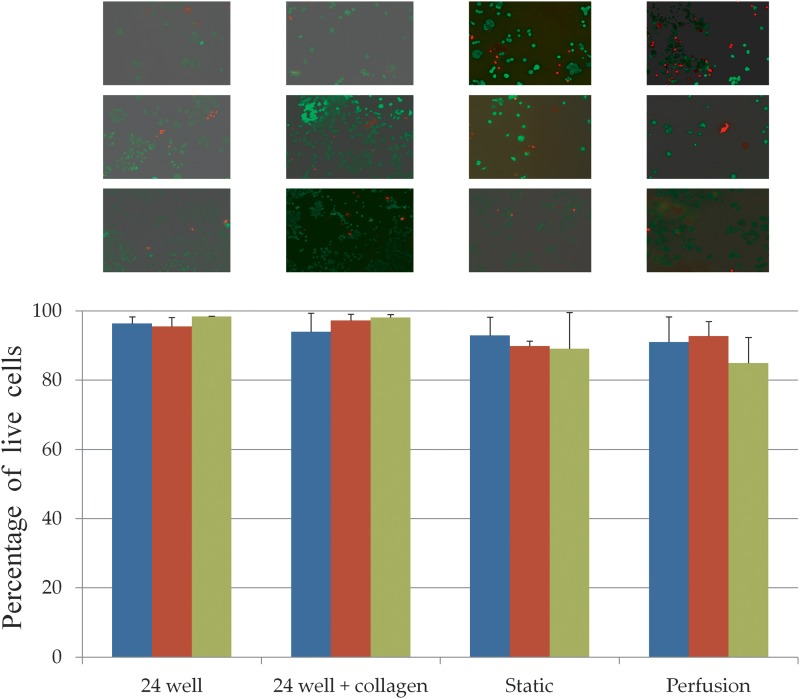
Viability of cells under perfusion in the bioreactor. Different bars represent different days of culture. Blue, day 2; red, day 4; green, day 6. The days are counted from the day when cells were seeded. Percentage of live cells in 24-well plate, 24-well plate coated with collagen, static and perfusion bioreactors are shown. Representative fluorescent images of each culture are shown above with the days increasing from top to bottom. Cells are stained using Calcein AM (live) and Ethidium Bromide (dead). All images were taken at 10× magnification.

Cell viability of MCF7 cells in perfusion bioreactor is compared with the cell viability in static bioreactor (i.e., no perfusion) in Figure 8. The cells were found to be viable under perfusion (>80%) even after 6 days without significant difference in cell viability from those in static bioreactors. The results demonstrate that the design and implementation of the perfusion culture system allows cell growth and culture. Interestingly, the absolute numbers of cells were lower in the bioreactors (both static and perfusion) in comparison with the 24-well plates, but the cell viability was similar (Fig. 8). This could be due to slower growth rate in the bioreactor. Since the cells in the bioreactors (static and perfusion) were grown on top of collagen-coated cover-slips, we wanted to rule out the effect of collagen on the cell viability. To investigate the effect of collagen coating on cell viability, we have performed the live–dead assay on the cells cultured in 24-well plates with and without collagen coating. The cell viability or total numbers of cells were not different between 24-well plates with and without collagen coating (Fig. 8). This shows that collagen does not affect the viability of MCF7 cells. Cell growth under perfusion for longer durations will be addressed in future experiments.

### Live imaging of NIH 3T3 mouse fibroblast cells on SU-8 scaffolds

**FIG. 9.**
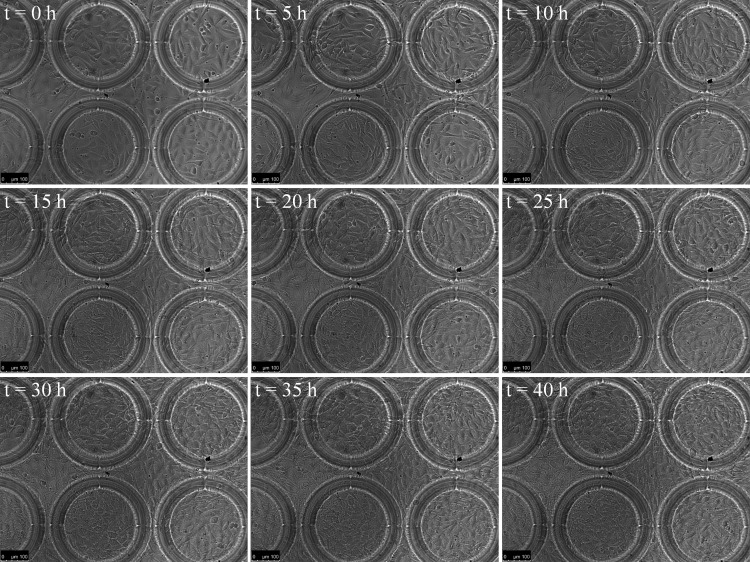
Live imaging of NIH 3T3 fibroblasts growing on ring scaffolds.

Microfabrication is a popular choice for making scaffolds of exact geometries for growing cells.^17,18^ Microfabricated scaffolds have been used for 3D cell culture,^19^ studying cell migration,^20^ etc. In this study, we demonstrate live imaging of NIH 3T3 mouse fibroblast cells (Fig. 9) on SU-8 ring scaffolds. There is no special significance about the ring geometry here; this readily available geometry is representative of many other geometries that can be fabricated with SU-8. Figure 9 shows that such microfabricated scaffolds can be conveniently inserted into our bioreactor and the effect of the scaffold on the cells can be studied. As can be observed from Figure 9, the cells gradually populated the entire scaffold and by the end of 40 h they had grown to almost full confluency. Cells were found to grow on the glass substrate inside the rings as well as on top of the rings.

The current study shows that microfabricated scaffolds can be inserted into the bioreactor and that cells can be grown on them conveniently. Many researchers use microfabricated structures as moulds for making soft polymer (like PDMS) scaffolds.^19^ In such cases, the final soft polymer scaffold can be attached to a cover-slip by oxygen plasma treatment and then inserted into our bioreactors. Once inserted into the bioreactors high-resolution live cell imaging and/or perfusion of media can be performed on the cells growing on these scaffolds.

### Primary rat hepatocytes in the bioreactor

**FIG. 10.**
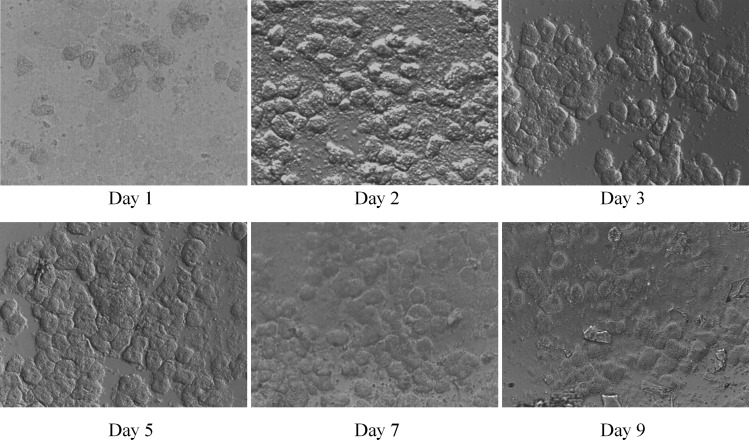
Primary rat hepatocytes in the static bioreactor.

The versatility of our bioreactor was further assessed by growing primary rat hepatocytes in them. Hepatocytes were found to be viable inside the bioreactor and could continue growing up to 9 days. After 9 days, the cells were apparently found to lose viability and were mostly in the suspension. The hepatocytes grew into cell aggregates and exhibited cuboidal and polygonal morphology inside the bioreactor, as has been reported in various previous studies^5,21^ (Fig. 10).

While many previously reported bioreactor systems (reviewed in Shulman and Nahmias^22^) have achieved longer term hepatocyte cultures, we have just shown that the basic miniature bioreactor that we have developed also supports primary cell culture. Long-term cell survival can be achieved by employing scaffolds, perfusion of media, and various other techniques such as collagen sandwich, etc., all of which can be conveniently incorporated into our system. In addition, our system can also be used for high-resolution live cell imaging. This could be useful for cell migration assays, drug toxicity studies, characterizing cell response to chemical stimuli, etc.

### High-magnification live imaging of HuH7 cells

**FIG. 11.**
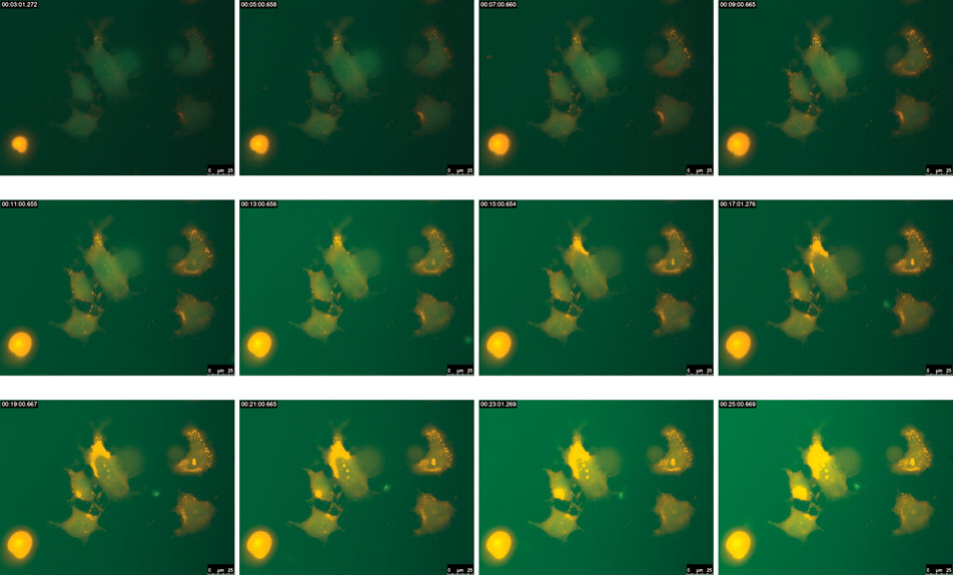
High-magnification time-lapse imaging of HuH7 cells incubated with live–dead assay mixture (Calcein AM–Ethidium Bromide).

Time-lapse images of the HuH7 cells incubated with the live–dead assay mixture are shown in Figure 11. The cells were mounted on the microscope at room temperature (25°C) and hence the cells slowly started to die. This is clearly captured in the live imaging as the cells which were fluorescing green (Calcein AM–stains live cells) slowly started to fluoresce red (Ethidium Bromide–stains dead cells; Fig. 11).

The oil immersion objective used has a working distance of about 0.1 mm and cannot focus across standard Petri dishes and is designed for imaging across a cover-slip. Since our bioreactor uses a cover-slip for growing cells and is open from below, an objective lens can easily approach the bioreactor from the bottom for imaging.

## Conclusion

We have designed, fabricated, and tested a perfusion culture system consisting of miniature bioreactors and miniature peristaltic pumps for doing perfusion culture completely inside standard incubators. The bioreactors were made of VeroWhite material and fabricated by 3D printing. Our study shows that this material and fabrication technique is conducive for *ex vivo* cell culture. This is significant because any stable structure can be fabricated easily using 3D printing and can be used as tissue engineering scaffolds or containers for cell culture. Furthermore, the material can be autoclaved which helps in effective and convenient sterilization of the bioreactors.

Scaffolds made by microfabrication on cover-slips can be incorporated directly into the bioreactor and cells grown under perfusion on these scaffolds. The bioreactor is also suitable for high-magnification imaging, single cell imaging studies since the cells are grown on cover-slips and hence they can be viewed from below on an inverted microscope. We have demonstrated that the bioreactor is suitable for general and perfusion tissue culture studies by culturing primary (rat hepatocytes), immortalized (NIH 3T3), and cancerous (MCF7) cells. We have also demonstrated high-resolution imaging of liver carcinoma (HuH7) cells. Our system is amenable to live-cell imaging, studying the growth of cells on scaffolds, and performing long-term perfusion culture completely inside an incubator. Furthermore, it can be used for studying the response of cells to chemical stimulants and drugs at a single cell level (through high-magnification fluorescence imaging) and at a population level in long-term perfusion cultures.

## Abbreviations Used

3D: three-dimensional
DMEM: Dulbecco's modified Eagle's medium
PBS: phosphate-buffered saline
PDMS: polydimethylsiloxane
UV: ultraviolet
